# Factors Associated with Vaccination Completeness and Knowledge About Hepatitis B Among Undergraduate Nursing Students in Northeastern Brazil: A Cross-Sectional Study

**DOI:** 10.3390/vaccines14100881

**Published:** 2026-10-05

**Authors:** Danielle Nedson Rodrigues de Macêdo, Breno Dias de Oliveira Martins, Luiza Carolinda de Sousa, Emanoelle Fernandes Silva, João Felipe Tinto Silva, Gilney Guerra de Medeiros, Polyana Norberta Mendes, Matheus Sousa Marques Carvalho, Jefferson Abraão Caetano Lira, Cecília Natielly da Silva Gomes, Telma Maria Evangelista de Araújo, Rosilane de Lima Brito Magalhães

**Affiliations:** 1 Health Sciences Center, Federal University of Piauí, Teresina 64049-550, Brazil; 2 Postgraduate Program in Biotechnology in Human and Animal Science, State University of Ceará, Ceará 60020-181, Brazil

**Keywords:** hepatitis B, hepatitis B vaccines, knowledge, students nursing

## Abstract

Hepatitis B remains an important occupational risk for undergraduate nursing students, particularly due to potential exposure, making vaccination and infection knowledge essential for protection throughout their professional careers. Objective: This study aimed to analyze the factors associated with vaccination completeness and knowledge about hepatitis B among undergraduate nursing students in Northeastern Brazil. Methods: An analytical cross-sectional study was conducted with undergraduate nursing students from three campuses of a public university in Northeastern Brazil. Data were collected between October and December 2025 using validated instruments that assessed sociodemographic and academic characteristics, knowledge, and vaccination status through analysis of vaccination cards. Associations were assessed using multivariable binomial logistic regression, and results are shown as adjusted odds ratios and 95% confidence intervals. Results: Vaccination completeness (minimum of 3 doses of HepB vaccine) was observed in 74.3% of the undergraduate nursing students, while 57.9% demonstrated adequate knowledge about hepatitis B (minimum of 75% of assessment questions properly answered). The variables associated with lower odds of vaccination completeness were a family income lower than two minimum wages, not having taken the infectious diseases course, absence of training on hepatitis B, not having undergone rapid test for sexually transmitted diseases, and reported current use of illicit drugs. Regarding inadequate knowledge about hepatitis B, students who had not taken the course related to infectious diseases and those who had not undergone rapid tests for HIV, syphilis, and hepatitis B were more likely to have inadequate knowledge. In contrast, hepatitis B training and previous rapid testing were associated with lower likelihood of inadequate knowledge. Conclusions: A substantial proportion of nursing students was incompletely vaccinated, while most demonstrated a good level of knowledge about hepatitis B. The findings reinforce the need to strengthen immunization initiatives from the beginning of undergraduate education, particularly by improving access to vaccination and providing training on hepatitis B.

## 1. Introduction

Hepatitis B remains an occupational risk for undergraduate nursing students who, from the early years of their training, are exposed to hepatitis B virus in healthcare practice settings. Exposure to clinical environments increases the possibility of contact with blood, bodily fluids, and potentially contaminated materials, making the protection of these students a relevant issue during professional training [1,2,3].

In this context, vaccination is the primary preventive measure against hepatitis B among nursing students. In Brazil, hepatitis B vaccination has been included in the National Immunization Program (PNI) since the 1990s and is provided free of charge through the Unified Health System (SUS). Vaccination is routinely recommended from childhood, with catch-up vaccination available for unvaccinated individuals and specific recommendations for special groups. Although there is no specific vaccination schedule for undergraduate nursing students, vaccination is particularly relevant because of their potential exposure to biological materials during clinical training [4,5]. Studies conducted among undergraduate nursing students reinforce the importance of monitoring the vaccination status of this group, given the challenges associated with maintaining adequate protection during training and throughout their professional careers [3,6].

Despite vaccine availability, completion of the vaccination schedule remains variable among healthcare students, with studies conducted in Kenya and Turkey reporting vaccination coverage ranging from 20.2% to 86% [7,8]. In Vietnam, only 30% of healthcare students had a complete hepatitis B vaccination schedule, while a small proportion had undergone testing before beginning practical activities, highlighting vulnerabilities that remain present in this group [9]. Further studies conducted among nursing students have demonstrated gaps in hepatitis B vaccination, including low vaccination coverage and incomplete vaccination schedules, reinforcing the importance of monitoring vaccination status throughout professional training [3,6,10,11].

Knowledge about hepatitis B is essential for risk perception, adoption of preventive measures, and safe practice in healthcare services [12,13]. However, the literature indicates limitations in knowledge among healthcare students, particularly regarding transmission, prevention, and protective measures against infection [14,15,16], highlighting the importance of strengthening the transmission of knowledge and skills related to hepatitis B prevention and occupational protection throughout their academic and professional trajectories.

Vaccination completeness and knowledge about hepatitis B may be associated with different student characteristics. Studies have identified associations between vaccination and sociodemographic, academic, and behavioral factors, including academic year, family income, parental education, area of residence, occupation, sex, and age [7,8,17,18,19]. In addition, knowledge about the infection has also been associated with vaccination status, with vaccinated healthcare students demonstrating higher levels of knowledge compared with unvaccinated students [8,11].

Although hepatitis B vaccination is routinely recommended and provided through Brazil’s National Immunization Program from childhood, not all individuals reach adulthood with a complete and documented vaccination schedule. Evidence investigating, in an integrated manner, the factors associated with completion of the hepatitis B vaccination schedule and the level of knowledge about the infection among undergraduate nursing students remains limited, particularly in the Brazilian context. Identifying these factors may contribute to recognizing more vulnerable groups and supporting targeted health education strategies and monitoring of vaccination status throughout professional training.

This study is justified by its potential contribution to strengthening immunization initiatives and nursing education. The findings may contribute to the development of educational and public health strategies aimed at protecting students and improving the quality of healthcare, in alignment with the goal of eliminating viral hepatitis as a public health problem by 2030. In light of this gap, this study aimed to analyze the factors associated with vaccination completeness and knowledge about hepatitis B among undergraduate nursing students in Northeastern Brazil.

## 2. Materials and Methods

### 2.1. Study Design and Setting

This analytical cross-sectional study was conducted in accordance with the Strengthening the Reporting of Observational Studies in Epidemiology (STROBE) guidelines. The study was conducted at one campus in the state capital, Teresina (Capital campus), and two campuses in the interior of Piauí, Floriano (Interior 1) and Picos (Interior 2), all belonging to the Federal University of Piauí (UFPI), a public university in the Northeast region of Brazil. The campuses were selected because they offer an undergraduate nursing program and provide an appropriate academic setting for investigating knowledge about hepatitis B and vaccination completeness among undergraduate students. The nursing program is offered in person on a full-time basis and includes theoretical and practical activities and curricular internships in different healthcare settings. The curriculum includes a specific course on infectious diseases, offered in the fifth semester of the undergraduate nursing program, which comprises nine semesters at the capital campus and ten semesters at the other campuses. From the fourth semester onward, students also begin practical clinical activities, including hospital visits, supervised clinical training, and exposure to patients and healthcare settings. These activities provide opportunities for contact with clinical conditions and potential occupational exposure to blood and other biological materials.

### 2.2. Population and Sample

The study population consisted of 774 undergraduate students regularly enrolled in the Bachelor’s degree program in Nursing across the three selected campuses. The sample size was determined using proportional stratified sampling, considering the distribution of students at each campus. The sample size was estimated considering a finite population, a 95% confidence level, a 5% sampling error, and an expected proportion of 50% (this value was used because a proportion of 50% maximizes the variability of a dichotomous outcome and, consequently, yields the largest required sample size for a given confidence level and sampling error), resulting in a minimum sample of 463 undergraduate students. The sample of undergraduate nursing students was distributed proportionally according to the number of students enrolled at each campus. Participants were recruited by convenience sampling, according to accessibility during the data collection period and voluntary participation in the study. Undergraduate nursing students who were duly enrolled and aged 18 years or older were included. Students without a vaccination card, with their enrollment suspended, or who had withdrawn from their academic program during the data collection period were excluded. The final sample consisted of 409 students, including 158 (38.6%) from Floriano (Interior 1), 135 (33.0%) from Picos (Interior 2), and 116 (28.4%) from Teresina (Capital campus). All eligible undergraduate nursing students in the participating classes were approached. Of the 774 students approached, 32 declined to participate (the reported reason was that they perceived the time required to complete the questionnaire as too long), resulting in 742 participants and a participation rate of 95.9% (742/774). Among these participants, 409 (55.1%) provided vaccination documentation and were included in the final sample, while 333 (44.9%) did not provide vaccination documentation and were excluded. Thus, the final sample consisted of 409 undergraduate nursing students.

### 2.3. Study Variables

The dependent variables of the study were hepatitis B vaccination completeness and knowledge about hepatitis B. The variable hepatitis B vaccination completeness corresponded to the vaccination status of the undergraduate students, determined by the number of doses documented on the vaccination card. According to the recommendations of the Brazilian National Immunization Program (Programa Nacional de Imunizações, PNI) [4], the hepatitis B vaccination schedule comprises three doses. Therefore, vaccination completeness was considered “yes” when the participant had at least three documented doses of hepatitis B vaccine, including the birth dose and doses received later in life, regardless of the administration of booster or additional doses, and “no” when fewer than three doses were documented on the vaccination card. Thus, participants with three or more documented doses were considered to have a complete vaccination schedule. This variable was classified as a dichotomous nominal categorical variable.

Knowledge about hepatitis B was assessed using the score obtained from the administered instrument [20]. Participants were classified as having “good” knowledge when they answered ≥75% of the items correctly and as having “poor” knowledge when they answered <75% correctly, following the classification adopted [20]. For the statistical analysis, knowledge was treated as a dichotomous categorical variable.

The independent variables comprised sociodemographic characteristics (sex, age, marital status, family income (assessed as the total monthly income of the participant’s household and categorized as up to two Brazilian minimum wages or three or more minimum wages, using the minimum wage in effect during the data collection period as the reference), and parental education), academic characteristics (campus, taking the infectious diseases course, and training on hepatitis B—participation in hepatitis B training was defined as participation in educational or training activities on hepatitis B beyond the regular undergraduate nursing curriculum), and behavioral characteristics (sexual practices; condom use; history of sexually transmitted infections; rapid testing for HIV, syphilis, hepatitis B, and hepatitis C; and alcohol, tobacco, and other drug use—participants were classified as current users, never users, or previous users who no longer used the substance; among current users, frequency of use was also assessed).

### 2.4. Data Collection and Research Instrument

Data were collected in person between October and December 2025, following the signing of the Informed Consent Form (ICF). Two validated instruments were used for data collection. The first was developed and validated by Oliveira et al. [20] and was adapted for the present study by adding a variable on hepatitis B vaccination status to address the study objectives. The instrument collected sociodemographic and academic information and assessed knowledge about hepatitis B. The knowledge assessment included 25 items addressing liver function after diagnosis, early diagnosis and treatment, the first vaccine dose at birth, screening for coinfections, risk of chronic infection among newborns, liver cancer, clinical presentation of acute infection, progression to chronic infection, disease notification, preventive therapy according to the serological profile, hepatitis B immunoglobulin (HBIG) administration, AST as a marker, HBsAg detection, the silent acute phase of hepatitis B, anti-HB interpretation, factors associated with lower vaccine response, revaccination when anti-HB is non-protective, HBeAg interpretation, total anti-HBc interpretation, vertical and sexual transmission, breastfeeding, and rapid testing. Each item had one correct answer, indicated by an asterisk (*), and all items were equally weighted. The number of correct answers was summed to obtain the total knowledge score. Participants were classified as having “good” knowledge when they answered ≥75% of the items correctly and as having “poor” knowledge when they answered <75% correctly, following the classification adopted by the original instrument [20].

The second instrument, adapted from [21], was a validated instrument designed to assess sexual behavior and related practices among the participants. Data collection was conducted exclusively by the principal researcher through visits to classrooms previously scheduled with course coordinators and faculty members, at times that did not interfere with academic activities. Before data collection, the principal researcher contacted the class leaders to disseminate information about the study and data collection procedures, including the request that students bring their hepatitis B vaccination card to the scheduled session. The instruments were completed individually in printed form, ensuring the privacy, anonymity, and confidentiality of the information. In addition to administering the instruments, the hepatitis B vaccination card was checked to verify vaccination completeness, and only undergraduate students who provided proof of vaccination at the time of data collection were included.

### 2.5. Data Analysis

The data were coded, double-entered, and validated to identify inconsistencies and were analyzed using Jamovi software (version 2.3, The jamovi project, Sydney, Australia). Descriptive analyses included absolute and relative frequencies for categorical variables and measures of central tendency and dispersion for numerical variables.

Initially, knowledge about hepatitis B was considered an independent variable and was analyzed in association with vaccination completeness to assess the relationship between the level of knowledge and the vaccination status of the undergraduate students. Subsequently, knowledge about hepatitis B was considered a dependent variable to identify the factors associated with its level among the undergraduate students, considering sociodemographic, academic, and behavioral characteristics.

Associations between variables were assessed using Pearson’s chi-square test or Fisher’s exact test, according to the distribution of the data. The magnitude of the associations was estimated using the crude odds ratio (cOR) and corresponding 95% confidence intervals (95% CIs). Variables with *p* ≤ 0.20 in the bivariate analysis were included in multivariable binomial logistic regression models using the Enter method, estimating adjusted odds ratios (aORs) and 95% CIs. In the first multivariable model, vaccination completeness was considered the event of interest, with incomplete vaccination as the reference category. In the second model, knowledge about hepatitis B was considered the outcome, with poor knowledge as the event of interest and good knowledge as the reference category. Model quality was assessed using the Akaike Information Criterion (AIC), and multicollinearity was assessed using the Variance Inflation Factor (VIF). A significance level of 5% was adopted.

### 2.6. Ethical Considerations

The study was approved by the Research Ethics Committee of the Federal University of Piauí (approval number 7,851,338/2025).

## 3. Results

The final sample consisted of 409 undergraduate students who provided vaccination documentation. This represented a reduction of 11.7% from the minimum estimated sample size of 463 students (54 students). All eligible students from the participating classes were approached, leaving no additional students available for recruitment. There was a predominance of female students (78.5%), with a mean age of 21.4 years (SD = 3.2). Most participants reported being single (95.8%), non-White (68.7%), and having a family income of up to two minimum wages (71.2%). Regarding academic characteristics, 50.1% of the students had already taken the course on infectious diseases, and 50.9% reported having participated in training on the topic of hepatitis B (Table 1).

Regarding behavioral characteristics, reports of male partners (46.5%) and vaginal sexual practices (51.3%) predominated. More than half of the participants had already undergone a rapid test for sexually transmitted diseases (51.3%). Alcohol consumption was reported by 52.6% of the undergraduate students, with monthly consumption being the most frequent (43.3%). Regarding vaccination, 74.3% had a complete vaccination schedule, as shown on their vaccination cards (Table 1).

Regarding knowledge about hepatitis B, 57.9% demonstrated a good level of knowledge (≥75% correct answers). A high frequency of correct responses was observed for aspects related to diagnosis, treatment, and modes of transmission (88.5–99.3%), whereas the lowest percentages of correct answers were observed for questions about breastfeeding, rapid testing, and interpretation of anti-HBs (57.9–69.9%) (Table 2).

Vaccination completeness was associated with higher family income, having taken the Infectious Diseases course, participation in training on hepatitis B, undergoing any rapid test (testing for HIV, syphilis, and hepatitis B), and illicit drug use. In the multivariable analysis, undergraduate students with a lower family income (up to two minimum wages) had lower odds of vaccination completeness (aOR = 0.38; 95% CI: 0.21–0.68; *p* = 0.001). Likewise, not having taken the course related to infectious diseases (aOR = 0.51; 95% CI: 0.32–0.81; *p* = 0.005), not participating in training on hepatitis b (aOR = 0.52; 95% CI: 0.36–0.89; *p* = 0.005), not undergoing any rapid test (aOR = 0.56; 95% CI: 0.36–0.89; *p* = 0.014), and not having undergone HIV testing (aOR = 0.57; 95% CI: 0.36–0.90; *p* = 0.017), syphilis testing (aOR = 0.58; 95% CI: 0.37–0.93; *p* = 0.024), or hepatitis B testing (aOR = 0.51; 95% CI: 0.32–0.82; *p* = 0.006) were associated with lower odds of vaccination completeness. Current illicit drug use also was associated with lower odds of vaccination completeness (aOR = 0.17; 95% CI: 0.03–0.94; *p* = 0.043) (Table 3).

Regarding inadequate knowledge about hepatitis B, students who had not taken the Infectious Diseases course and those who had not undergone rapid tests for HIV, syphilis, and hepatitis B were more likely to present inadequate knowledge. Conversely, participation in hepatitis B training and having undergone rapid testing were associated with lower odds of inadequate knowledge.

In the multivariate analysis, academic and behavioral variables remained associated with knowledge about hepatitis B. Not having taken the course related to infectious diseases was significantly associated with poor/inadequate knowledge about hepatitis B (aOR = 0.47; 95% CI: 0.32–0.71; *p* < 0.001). Similarly, not having undergone rapid testing for HIV (aOR = 0.61; 95% CI: 0.41–0.90; *p* = 0.015), syphilis (aOR = 0.59; 95% CI: 0.39–0.87; *p* = 0.009), and hepatitis B (aOR = 0.65; 95% CI: 0.43–0.96; *p* = 0.031) remained associated with poor/inadequate knowledge about hepatitis B. On the other hand, having participated in hepatitis B training (aOR = 2.21; 95% CI: 1.47–3.39; *p* < 0.001) and having undergone rapid testing (aOR = 1.58; 95% CI: 1.06–2.34; *p* = 0.024) remained associated with lower odds of inadequate knowledge about hepatitis B (Table 4).

## 4. Discussion

This study found that approximately one-quarter (25.7%) of undergraduate nursing students had an incomplete hepatitis B vaccination schedule, while a substantial proportion demonstrated inadequate knowledge about the infection (42.1%). The main factors associated with vaccination completeness were higher family income; having taken the course related to infectious diseases; having undergone rapid tests for HIV, syphilis, and hepatitis B; and current illicit drug use. Regarding knowledge of hepatitis B, academic factors, such as taking the course related to infectious diseases and participating in training on hepatitis B, as well as undergoing rapid tests for sexually transmitted infections, stood out positively.

The age profile of the participants suggests a predominantly young and female group, with high dependence on family income. This profile is similar to that described in studies conducted with nursing and health sciences students in different contexts, in which young individuals at the beginning of their academic trajectory and influenced by their family socioeconomic context predominated [8,13,17,22].

Notably, unlike what has been observed in other studies, parental education was not associated with hepatitis B vaccination completeness in this study. Other studies involving undergraduate nursing students showed that higher parental education was associated with a greater likelihood of adopting preventive practices related to the disease [13,17]. This discrepancy may reflect differences in the socioeconomic and educational contexts of the populations investigated.

Rapid testing was reported by slightly more than half of the undergraduate students (51.3%), with hepatitis B testing being less frequent than HIV and syphilis testing. Considering the young profile of the study population, this proportion represents a relatively high level of testing and indicates that a considerable proportion of students had already accessed rapid testing services. Nevertheless, nearly half of the students (48.7%) had not undergone any rapid testing, representing an important missed opportunity for early diagnosis, counseling, and preventive care. This finding should be interpreted in the context of national and international studies on testing practices, which have documented gaps in access to and uptake of testing, particularly among populations at risk of sexually transmitted infections [23,24].

Illicit drug use was infrequent (1.5%) among the undergraduate students, whereas alcohol consumption was reported by a substantial proportion (52.6%) of the sample. This profile is similar to that observed in studies of university students, in which alcohol consumption tends to be more frequent than illicit drug use [25,26]. Although nursing education facilitates access to information on health promotion and disease prevention, risk behaviors may still be present among university students, reinforcing the importance of ongoing health education initiatives throughout undergraduate training.

Hepatitis B vaccination completeness was high among (74.3%) undergraduate nursing students; however, a substantial proportion of students with an incomplete vaccination schedule persisted. The proportion observed in this study is consistent with studies conducted in different countries, which demonstrate wide variation in vaccination coverage among health sciences students [8,18,19,22,23,24]. From this perspective, the proportion of undergraduate students without a complete hepatitis B vaccination schedule highlights a relevant gap in student protection throughout undergraduate training.

The relevance of vaccination completeness among undergraduate nursing students becomes even more evident when considering the occupational risk inherent in practical activities during undergraduate training. Evidence demonstrates a significant occurrence of needlestick and other sharps injuries among undergraduate nursing students [1,2]. The presence of incomplete vaccination may represent an additional vulnerability, reinforcing the importance of institutional strategies aimed at verifying vaccination status throughout training.

This study showed that most (57.9%) undergraduate students had a good level of knowledge about hepatitis B; however, a substantial proportion still had insufficient knowledge about the infection. Investigations conducted among other health sciences students also demonstrate that, despite the predominance of good levels of knowledge, an important proportion of undergraduate students remains insufficiently knowledgeable about the infection [14,27].

Adequate knowledge was predominantly observed in aspects central to clinical practice, such as early diagnosis, modes of transmission, severity of the infection, and preventive measures, corroborating international studies [6,8,9]. However, knowledge was not uniform across the different topics assessed, with gaps persisting in specific areas, a finding similar to that described in national and international studies involving undergraduate nursing students [17,27,28]. The identified gaps indicate the need to further address the clinical and epidemiological aspects of hepatitis B during undergraduate training, promoting a more comprehensive understanding of the infection.

The association between family income and hepatitis B vaccination completeness reinforces the influence of socioeconomic factors on the adoption of preventive measures. Although the National Immunization Program (PNI) provides the recommended vaccines free of charge, barriers related to living conditions, such as transportation difficulties, scheduling incompatibility, and limited access to health information, may hinder the use of health services and maintenance of a complete vaccination schedule [29].

Similar results have been described in studies conducted in different countries, which also identified an association between better socioeconomic conditions and greater adherence to hepatitis B vaccination among health sciences students [7,8,11,30]. Although some of these studies use parental education as an indicator, this factor often reflects better economic conditions and greater access to health resources, corroborating the influence of the socioeconomic context observed in this study.

In the academic context, not having taken the course related to infectious diseases and not participating in training on the topic remained associated with higher odds of incomplete vaccination. These findings suggest that academic education and continuing education activities may promote greater awareness of occupational risks, the importance of immunization, and vaccination recommendations for health sciences students. Considering that the course is offered in the fifth academic period, it is possible that students who have already taken it also have greater exposure to practical activities, curricular internships, and health services, increasing opportunities for guidance and vaccination schedule updates.

Similarly, studies conducted in Somalia and Ghana demonstrated better vaccination indicators and preventive practices among students in more advanced stages of undergraduate training [13,31]. Although knowledge about hepatitis B was not associated with vaccination completeness in this study, the association observed for the course and training suggests that educational experiences and the university environment play a relevant role in vaccination adherence [8]. These findings reinforce the importance of promoting updates to the hepatitis B vaccination schedule from the early stages of undergraduate training, before the start of practical activities.

Adherence to the vaccination schedule was not directly associated with the presence of most risk behaviors but depended on other factors. International investigations involving health sciences students indicate that vaccination completeness tends to be more closely related to educational factors, such as a higher level of knowledge and advancement in the course, than to individual risk behaviors [31,32].

The association between illicit drug use and lower vaccination completeness is a finding that has been scarcely described in the literature on hepatitis B vaccination among health sciences students. This behavior may be related to lower adherence to preventive measures and lower use of health services; however, this hypothesis cannot be confirmed by the data from this study. International studies indicate that attitudes, preventive practices, and perceived vulnerability directly influence adherence to the vaccination schedule among health sciences students [31,32], reinforcing that vaccination is influenced by multiple individual, behavioral, and contextual factors.

Greater contact with health services and prevention activities may favor adherence to the hepatitis B vaccination schedule. This interpretation is supported by the observed association between not undergoing a rapid test and not undergoing HIV, syphilis, and hepatitis B testing and lower odds of vaccination completeness. Similarly, a study conducted among nursing students in the Attica region of Greece demonstrated an association between vaccination adherence, access to health services, and engagement in preventive practices [32], reinforcing the potential of these services to integrate testing, counseling, and vaccination schedule updates.

Regarding the level of knowledge, sex, age, skin color, parental education, income, and employment status were not associated with the outcome knowledge of HepB. This finding differs from the literature, which indicates an influence of socioeconomic context and family educational capital on health knowledge and practices [8,13]. In the present study, the findings suggest a relatively homogeneous distribution of knowledge among students, regardless of their social conditions, suggesting that factors related to academic training may be more relevant to knowledge about hepatitis B than some individual sociodemographic characteristics.

In this context, nursing education plays a central role in the development of knowledge about hepatitis B. Students who had not taken the Infectious Diseases course had lower odds of adequate knowledge about hepatitis B, corresponding to approximately 2.1 times higher odds of inadequate knowledge. In contrast, students who had participated in hepatitis B training had 2.21 times higher odds of adequate knowledge. These findings corroborate studies demonstrating greater knowledge among students exposed to courses and training on hepatitis B and other vaccine-preventable diseases [8,13,15,27].

In addition to academic training, participation in preventive activities was also as-sociated with knowledge about hepatitis B. A similar pattern was observed among those who had not undergone HIV, syphilis, and hepatitis B testing. One possible explanation is that contact with testing services provides additional opportunities for counseling and health education. A study conducted among Brazilian university students demonstrated that contact with testing and counseling services increases access to information about sexually transmitted infections [23].

### Study Limitations

The main limitation of this study was the use of convenience sampling, based on participant accessibility, which may have introduced selection bias and limits the generalizability of the findings. Students who were more accessible and available to participate during the data collection period may have differed from those who were not recruited, particularly regarding adherence to the hepatitis B vaccination schedule and exposure to academic activities related to the infection. If these characteristics were more common among participating students, the observed levels of vaccination completeness and knowledge may have been overestimated. In addition, the observed association between family income and hepatitis B vaccination completeness should be interpreted with caution, as self-reported family income may have been subject to misclassification. The exclusion of participants without vaccination documentation may also have affected the representativeness of the sample.

The smaller-than-planned sample size may have reduced the statistical power to identify additional associations. All eligible students from the participating classes were approached; however, no additional eligible students were available for recruitment after the predefined eligibility criteria were applied.

Despite this limitation, the findings provide relevant information on factors associated with hepatitis B vaccination completeness among undergraduate nursing students and may contribute to the development of strategies to strengthen vaccination in university settings.

## 5. Conclusions

This study demonstrated that, although most undergraduate nursing students had a complete hepatitis B vaccination schedule and adequate knowledge about the infection, a substantial proportion still had incomplete vaccination and inadequate knowledge. Incomplete vaccination was associated with socioeconomic, academic, and behavioral factors, particularly lower family income, not having taken the Infectious Diseases course, absence of hepatitis B training, and not having previously undergone rapid testing. Illicit drug use was also associated with incomplete vaccination.

Regarding knowledge about hepatitis B, academic training and preventive healthcare experiences were associated with the outcome. Not having taken the Infectious Diseases course and not having undergone rapid testing for HIV, syphilis, and hepatitis B were associated with inadequate knowledge, whereas participation in hepatitis B training and having undergone rapid testing were associated with lower odds of inadequate knowledge. These findings highlight the importance of integrating formal academic training with opportunities for health education and preventive care throughout undergraduate nursing education.

Therefore, institutional strategies should integrate vaccination status verification, immunization schedule updates, hepatitis B education, and access to preventive services during undergraduate training. Such actions may contribute to improving vaccination coverage and knowledge among future nursing professionals and reducing their occupational vulnerability to hepatitis B virus infection.

## Figures and Tables

**Table 1:** Characterization of undergraduate nursing students. Teresina, Piauí, Brazil, 2025 (*n* = 409).

Variable	*n*	%
Sex		
Male	88	21.5
Female	321	78.5
Age (years)		
Mean (standard deviation)	21.4 (3.2)	-
Median (Minimum–Maximum)	21.0 (18.0–47.0)	-
Marital status		
Single	392	95.8
Married or in a stable union	15	3.7
Divorced/separated	2	0.5
Skin color		
White	128	31.3
Non-White	281	68.7
Parental education (years of schooling)		
≤9 years	91	22.2
10–12 years	163	39.9
≥13 years	155	37.9
Family income		
Up to 2 minimum wages	291	71.2
3 minimum wages or more	118	28.8
Campus		
Capital campus	116	28.4
Interior campus 1	158	38.6
Interior campus 2	135	33.0
Took the Infectious Diseases course		
Yes	205	50.1
No	204	49.9
Number of sexual partners in the past 12 months		
Mean (Standard Deviation)	1 (1.7)	-
Median (Minimum–Maximum)	1 (0–16)	-
Types of sexual partners		
Men	190	46.5
Women	41	10.0
Men and women	3	0.7
Not applicable	175	42.8
Type of sexual practice		
Anal		
Yes	33	8.1
No	376	91.9
Vaginal		
Yes	210	51.3
No	199	48.7
Oral		
Yes	105	25.7
No	304	74.3
Use of Sex toys		
Yes	8	2.0
No	401	98.0
Ever had an STI/genital tract infection		
Yes	53	12.9
No	339	82.9
Do not know	17	4.2
Ever undergone a rapid test		
Yes	210	51.3
No	199	48.7
Type of rapid test		
HIV		
Yes	193	47.2
No	216	52.8
Syphilis		
Yes	183	44.7
No	226	55.3
Hepatitis B		
Yes	180	44.0
No	229	56.0
Hepatitis C		
Yes	161	39.4
No	248	60.6
Alcohol use		
Yes	215	52.6
No	158	38.6
Previously used, but no longer uses	36	8.8
Frequency of alcohol consumption (*n* = 215)		
Weekly	37	17.2
Monthly	93	43.3
Annually	85	39.5
Tobacco use		
Yes	8	2.0
No	384	93.8
Previously used, but no longer uses	17	4.2
Frequency of tobacco use (*n* = 8)		
Monthly	3	37.5
Annually	5	62.5
Illicit drug use		
Yes	6	1.5
No	396	96.8
Previously used, but no longer uses	7	1.7
Frequency of illicit drug use (*n* = 6)		
Occasionally	6	100
Hepatitis B vaccination completeness		
Yes	304	74.3
No	105	25.7

**Table 2:** Characterization of knowledge about hepatitis B among undergraduate nursing students. Teresina, Piauí, Brazil, 2025 (*n* = 409).

Question/Answer	*n*	%
Assessment of liver function after diagnosis		
Correct *	402	98.3
Incorrect	7	1.7
Early diagnosis allows appropriate treatment		
Correct *	406	99.3
Incorrect	3	0.7
Treatment goal: reduce disease progression		
Correct *	392	95.8
Incorrect	17	4.2
First vaccine dose at birth		
Correct *	366	89.5
Incorrect	43	10.5
Screening for coinfections		
Correct *	335	81.9
Incorrect	74	18.1
Newborns are at risk of chronic hepatitis B		
Correct *	355	86.8
Incorrect	54	13.2
Hepatitis B causes liver cancer		
Correct *	287	70.2
Incorrect	122	29.8
Acute infection can be asymptomatic or severe		
Correct *	343	83.9
Incorrect	66	16.1
5–10% develop chronic infection		
Correct *	355	86.8
Incorrect	54	13.2
Hepatitis B is a notifiable disease		
Correct *	307	75.1
Incorrect	102	24.9
Preventive therapy guided by the serological profile		
Correct *	352	86.1
Incorrect	57	13.9
HBIG within the first 12–24 h		
Correct *	347	84.8
Incorrect	62	15.2
AST as a sensitive marker		
Correct *	310	75.8
Incorrect	99	24.2
HBsAg detected in the acute and chronic phases		
Correct *	313	76.5
Incorrect	96	23.5
Silent hepatitis B in the acute phase		
Correct *	301	73.6
Incorrect	108	26.4
Anti-HB ≥10 IU/mL indicates immunity		
Correct *	267	65.3
Incorrect	142	34.7
Factors associated with a lower response to vaccination		
Correct *	295	72.1
Incorrect	114	27.9
Repeat the vaccination schedule if anti-HB is non-protective		
Correct *	276	67.5
Incorrect	133	32.5
HBeAg indicates infection with a high viral load		
Correct *	308	75.3
Incorrect	101	24.7
Total anti-HBc indicates infection		
Correct *	271	66.3
Incorrect	138	33.7
Vertical transmission		
Correct *	362	88.5
Incorrect	47	11.5
Sexual transmission		
Correct *	372	91.0
Incorrect	37	9.0
Transmission through breastfeeding		
Correct	237	57.9
Incorrect *	172	42.1
Rapid test is diagnostic		
Correct	239	69.9
Incorrect *	170	41.6
HBsAg is not always detected in the acute phase		
Correct *	286	69.9
Incorrect	123	30.1
Correct answers		
Good/Adequate (≥75% correct answers)	237	57.9
Poor/Inadequate (<75% correct answers)	172	42.1

* Legend: Correct answer.

**Table 3:** Multivariable binomial logistic regression of factors associated with hepatitis B vaccination completeness. Teresina, Piauí, Brazil, 2025 (*n* = 409).

Variables ^a^	Hepatitis B Vaccination Completeness				Bivariate Analysis Crude OR (95% CI)	*p*-Value	Multivariable Analysis Adjusted OR (95% CI)	*p*-Value ^b^
	Yes		No					
	*n*	%	*n*	%				
Parental education (years of schooling)					**-** ^c^	0.191 ^d^		
≤9 years	65	71.4	26	28.6			0.65 (0.35–1.18)	0.159
10–12 years	116	71.2	47	28.8			0.64 (0.38–1.08)	0.092
≥13 years	123	79.4	32	20.6				
Family income					2.58 (1.45–4.56)	<0.001 ^d^		
Up to 2 minimum wages	203	69.8	88	30.2			0.38 (0.21–0.68)	0.001
3 minimum wages or more	101	85.6	17	14.4				
Took the Infectious Diseases course					0.51 (0.32–0.81)	0.004 ^d^		
Yes	165	80.5	40	19.5				
No	139	68.1	65	31.9			0.51 (0.32–0.81)	0.005
Training on hepatitis B					1.90 (1.21–2.99)	0.005 ^d^		
Yes	167	80.3	41	19.7				
No	137	68.2	64	31.8			0.52 (0.36–0.89)	0.005
Ever undergone a rapid test					1.76 (1.12–2.76)	0.013 ^d^		
Yes	167	79.5	43	20.5				
No	137	68.8	62	31.2			0.56 (0.36–0.89)	0.014
Type of rapid test								
HIV					0.57 (0.36–0.90)	0.017 ^d^		
No	150	69.4	66	30.6			0.57 (0.36–0.90)	0.017
Yes	154	79.8	39	20.2				
Syphilis					0.58 (0.37–0.93)	0.023 ^d^		
No	158	69.9	68	30.1			0.58 (0.37–0.93)	0.024
Yes	146	79.8	37	20.2				
Hepatitis B					0.51 (0.32–0.82)	0.005 ^d^		
No	158	69.0	71	31.0			0.51 (0.32–0.82)	0.006
Yes	146	81.1	34	18.9				
Hepatitis C					0.63 (0.39–1.01)	0.054 ^d^		
No	176	71.0	72	29.0			0.63 (0.39–1.01)	0.055
Yes	128	79.5	33	20.5				
Alcohol use					**-** ^c^	0.168 ^d^		
Yes	155	72.1	60	27.9			1.46 (0.90–2.38)	0.123
No	125	79.1	33	20.9				
Previously used, but no longer uses	24	66.7	12	33.3			0.77 (0.36–1.65)	0.506
Illicit drug use					**-** ^c^	0.025 ^e^		
Yes	2	33.3	4	66.7			0.17 (0.03–0.94)	0.043
No	295	74.5	101	25.5				
Previously used, but no longer uses	7	1.7	0	0			<0.001 (0.00–0.00)	0.979
Knowledge about hepatitis B					0.70 (0.44–1.09)	0.117 ^d^		
Good/Adequate (≥75% correct answers)	183	77.2	54	22.8				
Poor/Inadequate (<75% correct answers)	121	70.3	51	29.7			0.70 (0.44–1.09)	0.117

Legend: ^a^ Variable included in the model because of *p* ≤ 0.20 in the bivariate analysis; ^b^ binomial logistic regression, with assessment of the assumption of multicollinearity; ^c^ could not be calculated; ^d^ Chi-square test; ^e^ Fisher’s exact test.

**Table 4:** Multivariable binomial logistic regression of factors associated with inadequate knowledge about hepatitis B. Teresina, Piauí, Brazil, 2025 (*n* = 409).

Variables ^a^	Knowledge About Hepatitis B				Bivariate Analysis Crude OR (95% CI)	*p*-Value	Multivariable Analysis Adjusted OR (95% CI)	*p*-Value ^b^
	Good/Adequate		Poor/Inadequate					
	*n*	%	*n*	%				
Marital status					**-** ^c^	0.083 ^d^		
Single	169	43.1	223	56.9				
Married/stable union	3	0.7	12	2.9			0.03 (0.09–1.18)	0.090
Divorced/separated	0	0	2	0.5			<0.001 (0.00–0.00)	0.982
Took the Infectious Diseases course					0.47 (0.32–0.71)	<0.001 ^e^		
Yes	68	33.2	137	66.8				
No	104	51.0	100	49.0			0.47 (0.32–0.71)	<0.001
Training on hepatitis B					2.21 (1.48–3.29)	<0.001 ^e^		
Yes	68	32.7	140	67.3			2.21 (1.47–3.29)	
No	104	51.7	97	48.3				<0.001
Type of sexual practice Anal					0.57 (0.26–1.24)	0.154 ^e^		
Yes	162	43.1	214	56.9			0.57 (0.26–1.24)	0.158
No	10	30.3	23	69.7				
Oral					0.64 (0.40–1.02)	0.061 ^e^		
Yes	136	44.7	168	55.3			0.64 (0.40–1.02)	0.062
No	36	34.3	69	65.7				
Ever undergone a rapid test					1.58 (1.06–2.34)	0.023 ^e^		
Yes	77	36.7	133	63.3			1.58 (1.06–2.34)	
No	95	47.7	104	52.3				0.024
Type of rapid test								
HIV					0.61 (0.41–0.90)	0.015 ^e^		
No	103	47.7	113	52.3			0.61 (0.41–0.90)	0.015
Yes	69	35.8	124	64.2				
Syphilis					0.59 (0.39–0.87)	0.009 ^e^		
No	108	47.8	118	52.2			0.59 (0.39–0.87)	0.009
Yes	64	35.0	119	65.0				
Hepatitis B					0.65 (0.43–0.96)	0.031 ^e^		
No	107	46.7	122	53.3			0.65 (0.43–0.96)	0.031
Yes	65	36.1	115	63.9				
Hepatitis C					0.69 (0.46–1.04)	0.074 ^e^		
No	113	45.6	135	54.4			0.69 (0.46–1.04)	0.075
Yes	59	36.6	102	63.4				

Legend: ^a^ Variable included in the model because of *p* ≤ 0.20 in the bivariate analysis. ^b^ Binomial logistic regression, with assessment of the assumption of multicollinearity. In the binomial logistic regression model, poor/inadequate knowledge was used as the reference category, and good/adequate knowledge was modeled as the outcome. Therefore, OR values greater than 1 indicate higher odds of good knowledge, whereas OR values lower than 1 indicate lower odds of good knowledge. ^c^ Could not be calculated; ^d^ Fisher’s exact test; ^e^ Chi-square test.

## Data Availability

The data presented in this study are available on request from the corresponding author due to ethical reasons and privacy data.
